# Identification and characterization of a novel stress-responsive outer membrane protein Lip40 from *Actinobacillus pleuropneumoniae*

**DOI:** 10.1186/s12896-015-0199-8

**Published:** 2015-11-25

**Authors:** Xuehe Hu, Hao Yan, Ke Liu, Jiansheng Hu, Chao Qi, Jihong Yang, Yanli Liu, Jin Zhao, Jinlin Liu

**Affiliations:** College of Life Sciences, Central China Normal University, Wuhan, Hubei 430079 China

## Abstract

**Background:**

*Actinobacillus pleuropneumoniae*, a Gram-negative bacterium, is the causative agent of porcine pleuropneumonia, a highly contagious and often fatal disease. Because current vaccines confer limited protection against *A. pleuropneumoniae* infection, the development of more effective vaccines is urgently required. The identification of immunogenic and protective antigens, such as an outer-membrane lipoprotein, will advance this purpose.

**Results:**

Sixty putative lipoproteins were predicted from the genomic sequence of *A. pleuropneumoniae* using multiple algorithms. Here, we focused on the characteristics of the putative lipoprotein Lip40 from *A. pleuropneumoniae* strain SLW01 (serovar 1). Lip40 shares sequence similarity with many bacterial lipoproteins, and the structural prediction of Lip40 suggests that it is similar to *A. pleuropneumoniae* TbpB. The *N*-terminus of Lip40 contains an interesting tandemly repeated sequence, Q(E/D/P)QPK. Real-time RT–PCR indicated that the expression of *lip40* was significantly upregulated at 42 °C, at 16 °C, and under anaerobic conditions. Recombinant Lip40 (rLip40) produced in *Escherichia coli* BL21(DE3) was specifically recognized by porcine convalescent serum directed against *A. pleuropneumoniae*. Lip40 was confirmed to localize at the bacterial outer membrane, and its expression was significantly stimulated when *A. pleuropneumoniae* was cultured under various stress conditions. Lip40 also protected 75 % of mice from fatal virulent *A. pleuropneumoniae* infection.

**Conclusions:**

The immunogenic outer-membrane protein Lip40 is stress responsive, protects mice against infection, and might be a virulence determinant. Further investigation of Lip40 should expedite vaccine development and provide insight into the pathogenesis of *A. pleuropneumoniae*.

**Electronic supplementary material:**

The online version of this article (doi:10.1186/s12896-015-0199-8) contains supplementary material, which is available to authorized users.

## Background

*Actinobacillus pleuropneumoniae* is the etiological agent of porcine pleuropneumonia, a severe and often fatal respiratory disease of swine, which is associated with significant economic losses in industrialized pig production worldwide [1]. To date, 16 serovars of *A. pleuropneumoniae* have been identified [2]. The pathogenesis of *A. pleuropneumoniae* infection is associated with several virulence factors, including but not limited to exotoxins. Other factors, such as capsular polysaccharides, lipopolysaccharides, adhesins, proteases, outer-membrane proteins, and transcriptional regulators, are also reported to be involved in its pathogenesis [3–6].

Bacterial lipoproteins are a set of membrane-associated proteins characterized by a conserved lipid-modified cysteine, which play important roles in bacterial physiological processes [7]. The involvement of lipoproteins in the infection processes of many pathogens has received wide attention [8]. Lipoproteins play a key role in the bacterium’s adhesion to the host cell, are involved in antibiotic resistance, and regulate the host immune response [8]. They have been reported to be pathogen-associated molecular patterns, which are recognized and captured by Toll-like receptors or other pattern-recognition receptors, inducing the activation of the immune cells and initiating the inflammatory processes [9]. Many immunogenic bacterial lipoproteins have been developed as candidate vaccines [8].

A putative lipoprotein, Lip40, containing the tandemly repeated sequence Q(E/D/P)QPK was observed among the 60 putative lipoproteins predicted from the published genomic sequence of *A. pleuropneumoniae* strain JL03. The *lip40* gene from an *A. pleuropneumoniae* field isolate, SLW01 (serovar 1), was cloned and characterized, including its sequence features, predicted structure, immunogenicity, subcellular localization, expression after stimulation, and protective efficiency in mice, extending our understanding of this lipoprotein.

## Methods

### Bacterial strains, plasmids, primers, and growth conditions

The bacterial strains, plasmids, and primers used in this work are listed in Table 1. The primers used in this study were synthesized by Sangon Biotech (Shanghai, China). *Escherichia coli* strains were cultured in Luria–Bertani broth, supplemented with the appropriate antibiotic (50 μg/ml ampicillin). *Actinobacillus pleuropneumoniae* strains were cultured in tryptic soy broth (TSB) or on tryptic soy agar (TSA) (Becton, Dickinson & Company, Franklin Lakes, New Jersey, USA), supplemented with 10 μg/ml nicotinamide adenine dinucleotide (Sigma-Aldrich Co., Ltd, St. Louis, Missouri, USA). For the anaerobic treatment, *A. pleuropneumoniae* was cultured under oxygen-free conditions (85 % N_2_, 10 % H_2_, and 5 % CO_2_) (Forma Anaerobic System, Thermo Fisher Scientific, Marietta, USA).

**Table 1:** Strains, plasmids, and primers used in this study

Strain, plasmid and primer	Relevant characteristics	Source
*A. pleuropneumoniae*		
SLW01	Serovar 1	Weicheng Bei
13039	Serovar 10	Pat Blackall
*E. coli*		
DH5a	Cloning vehicle: *supE44* Δ*lacU169* (*φ80 lacZ*ΔM15) *hsdR17recA1 endA1 gyrA96 thi-1 relA1*	Takara (Dalian, China)
BL21(DE3)	Expression host: F^−^ *ompT* r^−^_B_ m^−^_B_; DE3 is a λ derivative carrying *lacI* and T7 RNA polymerase genes under placUV5 control	Takara (Dalian, China)
Plasmid		
pMD18-T	*E. coli* cloning vector carrying an ampicillin resistance determinant	Takara (Dalian, China)
pMD-lip40	pMD18-T carrying *lip40* gene of *A. pleuropneumoniae* strain SLW01.	This work
pGEX-KG	*N*-terminal glutathione *S*-transferase (GST) fusion expression vector: pBR322 *ori*, Amp^r^	[19]
pGEX-lip40	pGEX-KG carrying the *lip40* gene for over-expression Lip40 protein	This work
Primers (Primers were all synthesized in Sangon, Shanghai, China)		
P1	5′ ATG AAA AAC ATC ACA AAA TTT GCA G 3′, upstream primer for *lip40* gene cloning	This work
P2	5′ TTA CTT TTG TTG TTT TGC GCC AAA 3′, downstream primer for *lip40* gene cloning	This work
P3	5′ CGG TTC GAT TTG GTG TGT ATG A 3′,upstream primer of *lip40* gene for qRT-PCR analysis	This work
P4	5′ AAC AAG TAA GCA TCA CCT GTG T 3′, downstream primer of *lip40* gene for qRT-PCR analysis	This work
P5	5′ AAG TGG CAG AGC TGG AAG AT 3′, upstream primer of internal control gene *rluC* for qRT-PCR analysis	This work
P6	5′ TCA CAC CAA AAC TCA AGC CG 3′, downstream primer of internal control gene *rluC* for qRT-PCR analysis	This work
P7	5′ TTG GAT CCT GTG GCA GTA AGA ACC ATT C 3′, upstream primer with *Bam*HI site (underlined) comprising positions 58 to 77 of *lip40* coding sequence	This work
P8	5′ GGA AGC TTT TAC TTT TGT TGT TTT GCG C 3′, downstream primer with *Hin*dIII site (underlined) comprising positions 878 to 897 of *lip40* coding sequence	This work

### Prediction of *A. pleuropneumoniae* lipoproteins

The previously sequenced and annotated *A. pleuropneumoniae* strain JL03 was selected to identify the lipoproteins of *A. pleuropneumoniae* [10]. The programs used for lipoprotein prediction were: (i) ScanProsite (http://prosite.expasy.org/) [11]; (ii) DOLOP (http://www.mrc-lmb.cam.ac.uk/genomes/dolop/) [12]; (iii) SignalP 4.1 (http://www.cbs.dtu.dk/services/SignalP/) [13]; (iv) PrediSi (http://www.predisi.de/) [14]; (v) Phobius (http://phobius.binf.ku.dk/) [15]; and (vi) LipoP 1.0 (http://www.cbs.dtu.dk/services/LipoP/) [16]. Possible lipoproteins were then subjected to a ‘majority vote’ selection procedure to exclude any false positive lipoproteins [17].

### Cloning and bioinformatic analysis of Lip40 protein

The genomic DNA was isolated from *A. pleuropneumoniae* SLW01 as described previously [18]. Specific primers for the *lip40* gene were designed according to the conserved features of the *lip40* sequence in the complete *A. pleuropneumoniae* genome. The *lip40* gene was cloned by PCR, and the product was inserted into pMD18-T (Takara, Dalian, China), generating the plasmid pMD-lip40. The fragment inserted into pMD-lip40 was sequenced in both directions. The sequence features were analyzed with LipoP 1.0. A multiple sequence alignment was constructed with BioEdit (version 7.0). The structure of Lip40 was predicted by with the SWISS-model method (http://swissmodel.expasy.org/), and the Lip40 protein was superimposed onto the N lobe and C lobe of TbpB (Protein Data Bank [PDB]: 3HOL) with WinCoot.

### Reverse transcription–real-time quantitative PCR (RT–qPCR)

Total RNA was extracted from an *A. pleuropneumoniae* culture with the RNeasy Mini kit (Qiagen, Shanghai, China), treated with DNase I (Invitrogen, CA, USA), and reverse-transcribed into cDNA with the Omniscript® RT Kit (Qiagen), according to the manufacturer’s protocol. The mRNA levels of *A. pleuropneumoniae* cultured under different conditions were measured with a SYBR Green-based method. The *rluC* gene was used as the internal control. qPCR was performed in a 20 μl reaction volume containing 10 μl of 2 × SYBR Green Realtime PCR Master Mix (Toyobo, Osaka, Japan), 1 μl of cDNA, 0.5 μl each of the forward and reverse primers (10 μmol/l), and 8 μl of dH_2_O. An ABI 7500 Sequence Detection System was used for the amplification reactions, and qPCR was performed in triplicate. The 2^–ΔΔCt^ method was used to calculate the relative expression of the *lip40* gene.

### Expression and purification of Lip40 from *E. coli*

The mature protein-coding sequence of Lip40 was cloned from the *A. pleuropneumoniae* genomic DNA using primers P7 and P8 (Table 1). The PCR products were then digested with *Bam*HI/*Hin*dIII and ligated into the prokaryotic expression vector pGEX-KG [19], generating the recombinant plasmid pGEX-lip40. Recombinant Lip40 (rLip40) was then expressed from pGEX-lip40 in *E. coli* BL21(DE3). rLip40 was collected from the *E. coli* lysate and purified with a glutathione (GST)–Sepharose 4B column (Amersham Biosciences, Buckinghamshire, England). The immunoreactivity of the purified rLip40 was verified with western blotting, using porcine convalescent serum directed against *A. pleuropneumoniae* as the primary antibody.

### Production of polyclonal antibodies against rLip40

Two female New Zealand white rabbits (~2.5 kg; purchased from the Centre for Disease Control and Prevention [CDC] of Hubei Province, China) were used to produce polyclonal antibodies directed against rLip40. All the animal experiments in the present study were approved by the Animal Experiment Committee of Central China Normal University. Each rabbit was first injected intradermally with purified rLip40 (650 μg). The purified protein (0.5 ml) was emulsified with an equal volume of Freund’s complete adjuvant (Sigma-Aldrich, USA). Animals were subsequently injected three times, at 15-day intervals, with the same immunogen emulsified with incomplete Freund’s adjuvant with the same regimen. Antisera were collected 10 days after the third booster immunization.

The titers and specificity of the polyclonal antibodies were evaluated with a Lip40 enzyme-linked immunosorbent assay (ELISA). Briefly, flat-bottomed 96-well polystyrene ELISA plates (Haimen Shengbang, Haimen, China) were coated with 0.17 μg of purified rLip40 diluted in 100 μl of coating buffer (50 mM sodium carbonate, pH 9.6). The coated plates were washed three times with phosphate-buffered saline (PBS) plus 0.05 % Tween 20 (PBST), blocked at 37 °C for 1 h with blocking buffer (5 % skimmed milk in PBST), and then washed three times with PBST. For the ELISA, 2 μl samples and 198 μl of PBST were added to each well in the first line of wells in the plate and 100 μl of PBST was added to the rest of the wells. The samples were diluted and incubated at 37 °C for 40 min. After four washes with PBST, 100 μl of horseradish peroxidase (HRP)-conjugated secondary antibody (Southern Biotechnology Associates, Birmingham, USA), diluted 1:5000 in PBST, was added to each well, and the plates were incubated at 37 °C for 30 min. After five washes, 100 μl of 3,3′,5,5′-tetramethylbenzidine color development solution (Biotime Biotech, Haimen, China) was added to each well. The plates were incubated at room temperature in the dark for approximately 10 min, and the catalytic reaction was then stopped with 50 μl of 1 % SDS. The optical density was read at 630 nm (OD_630_) in an ELISA reader (PowerWave XS, Bio-Tek, Winooski, USA).

### Subcellular localization of Lip40

The subcellular fractionation of *A. pleuropneumoniae* was performed as described previously [20]. In brief, 1 l of bacterial culture was pelleted and resuspended in 10 ml of solution A (0.2 M Tris–HCl [pH 8.0], 1 M sucrose, 1 mM EDTA). Lysozyme was then added to the cell suspension to a final concentration of 1 mg/ml, vortexed, and incubated at room temperature for 5 min. dH_2_O (40 ml) was added to the swirling mixture before it was placed on ice. The cells were centrifuged at 200,000 × *g* for 45 min at 4 °C. The supernatant contained the periplasmic fraction. The pellet was resuspended in 7.5 ml of ice-cold solution B (10 mM Tris–HCl [pH 7.5], 5 mM EDTA, 0.2 mM DTT), supplemented with 50 μl of DNase (1 mg/ml). The cells were broken by two passes at 10^8^ Pa. The unbroken cells were pelleted by centrifugation at 4000 × *g* for 10 min at 4 °C. The supernatant was then centrifuged at 280,000 × *g* for 4 h at 4 °C. The supernatant contained the cytoplasmic fraction and the pellet contained the crude membranes, which were collected separately. The crude membrane pellet was resuspended in 9 ml of solution C (50 mM Tris–HCl [pH 8.0], 2 % [*v/v*] Triton X-100, 10 mM MgCl_2_), and centrifuged at 85,000 × *g* for 30 min at 4 °C. The supernatant contained the cytoplasmic membrane fraction. The pellet, containing the outer membrane, was washed in 1 ml of solution C, centrifuged at 85,000 × *g* for 20 min at 4 °C, washed three times with 500 μl of dH_2_O, and stored at −20 °C. The extracellular proteins were precipitated from the filtered culture supernatant with trichloroacetic acid at 4 °C overnight, followed by centrifugation (17,700 × *g*, 1 h, 4 °C), and were washed six times with 1 ml of 96 % ethanol. The pellet was dried and redissolved in 400 μl of solution D (7 M urea, 2 M thiourea, 4 % CHAPS [*w/v*], 30 mM Tris–HCl, [pH 8.0]), and stored at −20 °C. The subcellular localization of the Lip40 protein was determined with western blotting using rabbit hyperimmune anti-rLip40 serum (1:400) as the primary antibody.

### Expression of Lip40 protein under stress conditions

*Actinobacillus pleuropneumoniae*, cultured in TSB under normal conditions (aerobically at 37 °C) for 3 h, was divided into four equal parts and incubated under anaerobic condition, or at 42 °C, 16 °C, 37 °C for another 3 h, respectively . The cells were harvested by centrifugation, and the outer-membrane fractions were extracted as described above. The concentration of all outer-membrane samples prepared under different culture conditions were determined with a BCA protein assay kit (GenStar BioSolutions, Beijing, China). Equal volumes of the samples (66 μg) were separated with 12 % sodium dodecyl sulfate polyacrylamide gel electrophoresis and transferred to a nitrocellulose membrane. Western blotting was performed with rabbit hyperimmune anti-rLip40 serum (1:400) as the primary antibody. The expression of Lip40 was determined in triplicate. The expression levels of Lip40 in the outer-membrane samples were determined according to the density of the bands, with the Quantity One software.

### Vaccination and challenge of mice

The protective efficacy of rLip40 was tested in a mouse vaccination/challenge model. Forty-eight 6-week-old specific-pathogen-free BALB/c mice (CDC, Hubei Province, China) were randomly allocated to four groups of 12 animals. ApxI toxin was obtained from the supernatant of *A. pleuropneumoniae* strain 13039 (serovar 10) cultures, as described previously [21], and inactivated with 0.4 % formaldehyde. Group 1 was inoculated with PBS and used as the negative control; group 2 was vaccinated with 150 μg of rLip40; group 3 was vaccinated with 150 μg of inactivated ApxI; and group 4 was vaccinated with a commercial trivalent inactivated vaccine (containing serovars 1, 2, and 7; purchased from Wuhan Keqian Biotech, China). For groups 2 and 3, the antigens were emulsified separately with complete Freund’s adjuvant for the first immunization, and with incomplete Freund’s adjuvant for the booster immunization. Serum samples were collected before each immunization and before the mice were challenged, and assayed with the Lip40-ELISA, as described above, and an ApxI-ELISA, as described previously [22]. Each group was intraperitoneally challenged with 2.5 × 10^6^ colony-forming units of log-phase *A. pleuropneumoniae* SLW01 14 days after the second vaccination. The numbers of surviving mice were recorded every day for 5 days after the challenge.

## Results

### Sixty putative *A. pleuropneumoniae* lipoproteins

With the ‘majority vote’ approach [17], 60 of the 89 identified putative lipoproteins from *A. pleuropneumoniae* JL03 were predicted as lipoproteins, including some previously reported lipoproteins, such as TbpB (gene locus APJL_0250) [23] and PalA (gene locus APJL_0317) [24], as well as several previously reported putative lipoproteins [25], indicating that our prediction method and results are reliable. The sequence characteristics of the 60 lipoproteins are listed in Additional file 1: Table S1. Putative lipoprotein Lip40, containing a special *N*-terminal tandemly repeated sequence, was further investigated in the present study.

### Lip40 has a conserved lipoprotein domain

**Fig. 1:**
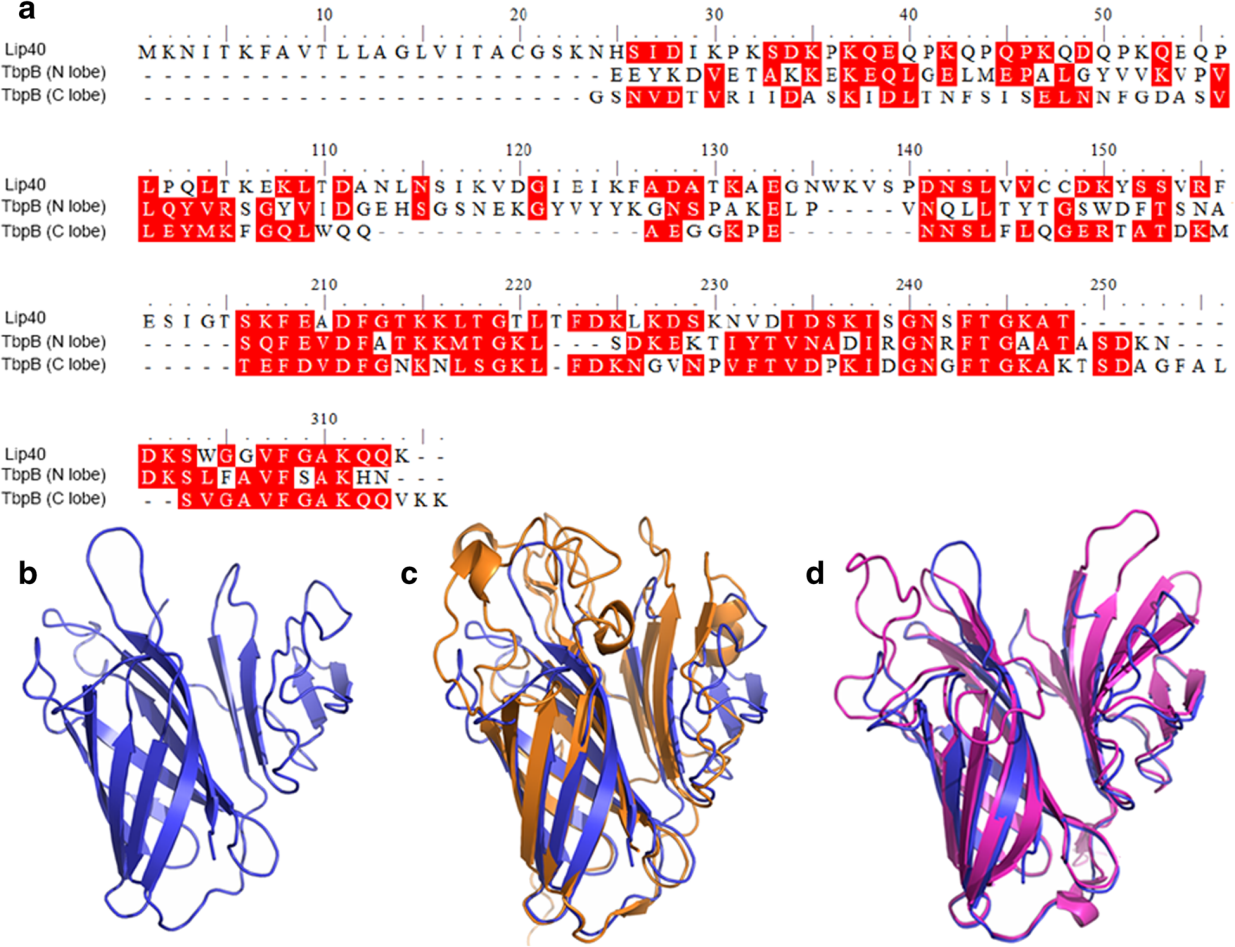
Sequence alignment and structural prediction of Lip40. **a** Sequence alignment of *A. pleuropneumoniae* Lip40 and the N- and C-lobes of *A. pleuropneumoniae* TbpB (PDB: 3HOL). **b** Overall structure of Lip40 protein (*blue*). The structure consists of an eight-stranded β barrel accompanied by a domain made up of four β strands. **c** Superimposition of Lip40 onto the TbpB N lobe (*orange*). **d** Superimposition of Lip40 onto the TbpB C lobe (*pink*)

The *lip40* gene from *A. pleuropneumoniae* SLW01 was cloned and sequenced (GenBank accession number: KP119767). The Lip40 protein was predicted to have the typical characteristics of lipoproteins with the LipoP 1.0 program, and contains the recognition sequence for signal peptidase II (LVITA^↓^CGSKN). The lipid modification site of Lip40 was predicted to be located at Cys20. A multiple sequence alignment of Lip40 and lipoproteins from *Aggregatibacter actinomycetemcomitans* and *Cardiobacterium hominis* showed that Lip40 is homologous to those lipoproteins (similarity > 33 %; Additional file 2: Figure S1). It also displayed similarity with several transferrin-binding proteins (Tbps) from pathogenic bacteria (similarity > 34 %; Additional file 3: Figure S2). The multiple sequence alignment showed that Lip40 is homologous to both the N- and C-lobes of *A. pleuropneumoniae* TbpB (Fig. 1a). Structural prediction revealed that the Lip40 protein consists of an eight-stranded β barrel accompanied by a domain made up of four β strands (Fig. 1b). Superimposition of Lip40 onto the C lobe and N lobe of TbpB (PDB: 3HOL) indicated that they have high structural similarity (Fig. 1c and d). We also noted that Lip40 contains a tandemly repeated sequence, Q(E/D/P)QPK, at its *N*-terminus. *Actinobacillus pleuropneumoniae* strain SLW01 Lip40 has nine tandem repeats. The numbers of repeats in the Lip40 homologues from other *A. pleuropneumoniae* strains were also calculated, and according to the available genome sequences of these strains, the repeat numbers differ among the different strains (8–27) (Table 2).

**Table 2:** Numbers of tandem repeats in different *A. pleuropneumoniae* strains

Strain	Serovar	Protein ID	No. of total amino acid	Tandem repeats		
				No. of start position	No. of end position	No. of repeats
SLW01	1	AKJ32477	298	38	82	9
4074	1	ZP_00134150.1	383	38	167	26
JL03	3	YP_001652178.1	378	38	162	25
M62	4	ZP_07532361.1	372	52	156	21
L20	5b	YP_001053852.1	293	38	77	8
Femo	6	ZP_07336294.1	368	38	152	23
AP76	7	YP_001969011.1	388	37	172	27
N273	13	ZP_07545435.1	397	52	181	26

### Transcription of *lip40* is stimulated by stress

**Fig. 2:**
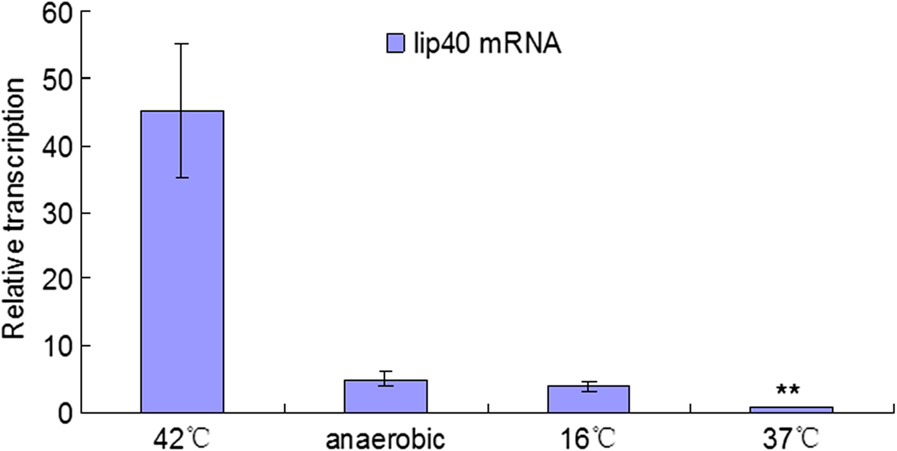
Transcription changes in the *A. pleuropneumoniae lip40* gene under different conditions. Relative expression of the *lip40* gene during stimulation with 42 °C, anaerobic conditions, or 16 °C was normalized to that of *lip40* expressed under normal culture conditions. The fold changes in *lip40* expression are shown. Error bars indicate standard errors. Double asterisks indicate *P* < 0.01, calculated with Student’s *t* test

The expression of the *lip40* gene was analyzed under *in vitro* growth conditions mimicking the environment during infection, including anaerobic conditions and high temperature (42 °C). The expression of *lip40* at low temperature (16 °C) was also evaluated. As shown in Fig. 2, the transcription of the *lip40* gene was significantly upregulated under stress relative to its transcription under normal culture conditions (37 °C, aerobic), and heat shock most strongly increased *lip40* expression (45-fold). These results indicate that *lip40* is responsive to temperature and anaerobic stress, and might be involved in the bacterial infection process.

### rLip40 is immunoreactive

**Fig. 3:**
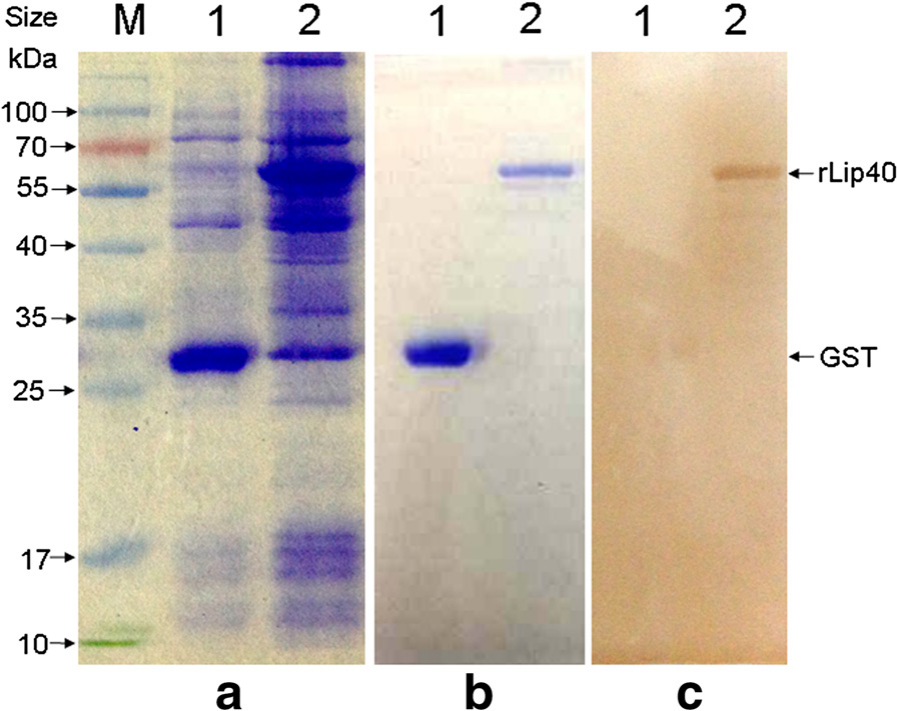
Expression (**a**), purification (**b**), and western blotting analysis (**c**) of rLip40 protein. Protein samples were separated with 12 % SDS-PAGE and blotted onto nitrocellulose membrane. The membrane was sequentially incubated with porcine convalescent serum directed against *A. pleuropneumoniae* and HRP-labeled goat anti-porcine IgG secondary antibody, and the color was developed with a 3,3′-diaminobenzidine (DAB) peroxidase substrate (Tiangen, Beijing, China). M, prestained protein ladder (Fermentas, Vilnius, Lithuania); lane 1, GST; lane 2, rLip40

To express the Lip40 protein, the *lip40* gene without the signal peptide was cloned from *A. pleuropneumoniae* SLW01, restricted, and ligated into the prokaryotic expression plasmid pGEX-KG. *Escherichia coli* cells containing pGEX-lip40 were induced with isopropyl-β-D-thiogalactopyranoside. The recombinant Lip40 protein was confirmed to be present in the soluble supernatant of the cell lysate with SDS-PAGE, as shown in Fig. 3a. The purification of rLip40 is shown in Fig. 3b. Western blotting revealed a band with a molecular weight of ~56 kDa for rLip40, whereas no signal was detectable for GST (Fig. 3c). This result confirms that porcine convalescent serum directed against *A. pleuropneumoniae* recognized and bound specifically to the rLip40 protein.

### Lip40 elicits a strong humoral immune response in rabbits

Rabbits were immunized with four doses of purified rLip40 by intradermal injection. Serum samples were collected before each immunization and before euthanasia and the antibodies against rLip40 were tested with an ELISA, to verify the generation of a Lip40-specific immune response in rabbits. Lip40-specific antibodies appeared two weeks after the first immunization, and then increased quickly after the second and third injections (Additional file 4: Figure S3A). At the end of the immunization regimen, titration of the rabbit sera (at 55 days) showed the highest reactivity, with an increase of up to 2 × 10^5^-fold (Additional file 4: Figure S3B), suggesting that rLip40 stimulates a strong antibody response in rabbits. The same amount of purified GST was used to coat 96-well plates, and antibody directed against GST was evaluated with a GST-ELISA, as described for the Lip40-ELISA. The GST-specific IgG titer in the hyperimmune serum was only 1:3200 (Additional file 4: Figure S3B), much lower than that of the Lip40-specific antibody. Therefore, it appears that Lip40 played a major role in the elicitation of rabbit antibodies directed against the GST–Lip40 fusion protein.

### Lip40 is located in the outer membrane

**Fig. 4:**
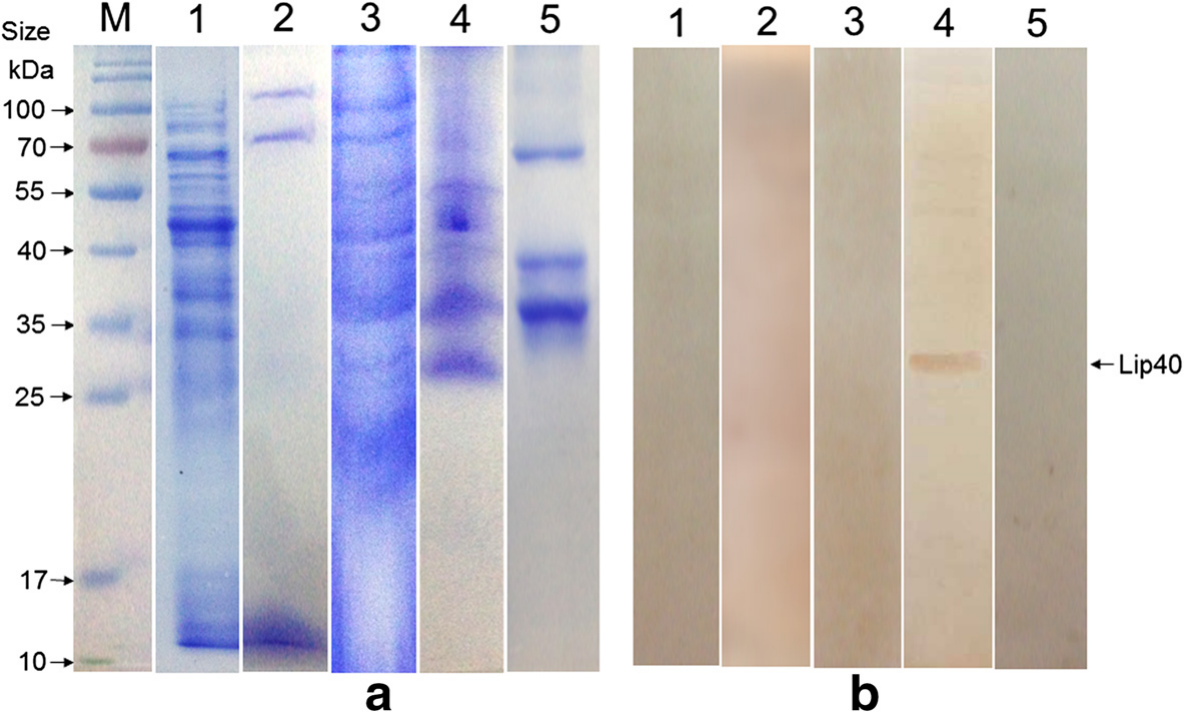
Subcellular localization of Lip40. **a** SDS-PAGE analysis of *A. pleuropneumoniae* subcellular fractions. **b** Western blotting analysis. Subcellular fractions were separated with 12 % SDS-PAGE, blotted onto nitrocellulose membranes, and incubated with rabbit anti-rLip40 polyclonal antibody. Color was developed with a DAB peroxidase substrate (Tiangen). M, prestained protein ladder; lane 1, cytoplasmic proteins (CP); lane 2, periplasmic proteins (PP); lane 3, cytoplasmic membrane proteins (CM); lane 4, outer-membrane proteins (OM); lane 5, extracellular proteins (EP). The nitrocellulose membranes displayed a band with a molecular weight of ~30 kDa in the outer-membrane fraction, indicating that Lip40 is located on the outer membrane. No signal was detected in the other subcellular fractions

Although bioinformatic analysis showed that Lip40 is located on the outer membrane, experimental verification was required (data not shown). Subcellular fractions of *A. pleuropneumoniae* cells were extracted carefully, including the cytoplasmic proteins, periplasmic proteins, cytoplasmic membrane proteins, outer-membrane proteins (OM), and extracellular proteins (Fig. 4a). A band corresponding to Lip40 (~30 kDa) was observed in the outer-membrane fraction with western blotting, as shown in Fig. 4b. No Lip40 signal was observed in the other subcellular fractions. Our results confirm that Lip40 localizes to the outer bacterial membrane.

### Enhanced expression of Lip40 protein under stress

**Fig. 5:**
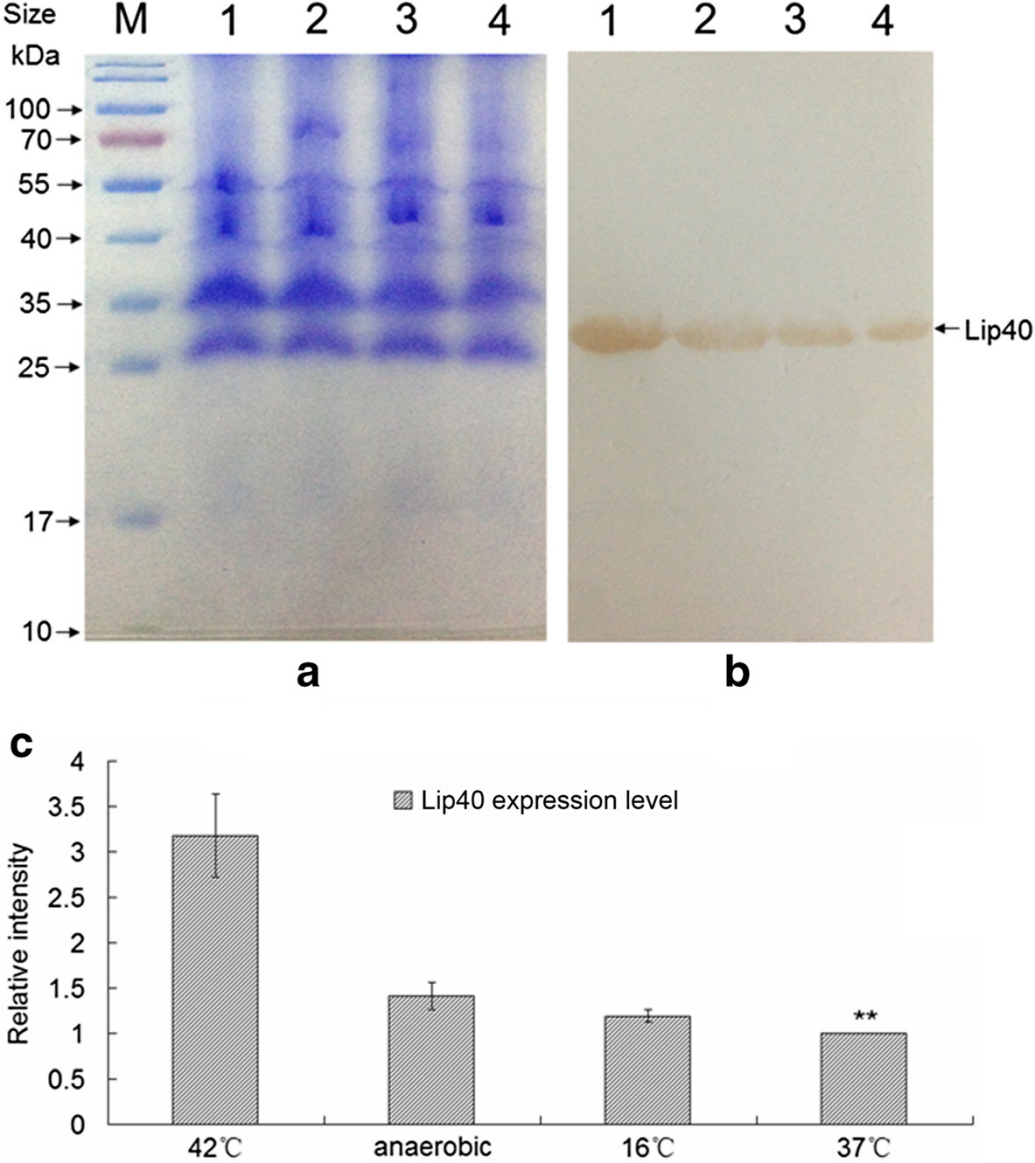
Expression changes of Lip40 protein in the outer-membrane fraction. **a** SDS-PAGE. *Actinobacillus pleuropneumoniae* outer-membrane samples were extracted from cells after they were treated with heating, anaerobic conditions, or cold stimulation, or with normal conditions. Equal amounts (66 μg) of outer-membrane proteins were analyzed with SDS-PAGE. **b** Western blotting analysis. The procedures were as described above. M, prestained protein ladder; lane 1, high temperature (42 °C); lane 2, anaerobic conditions; lane 3, low temperature (16 °C); lane 4, normal conditions (37 °C). **c** Intensity of the bands was quantified with the Quantity One software. Relative expression levels were obtained by normalizing the intensity of stress-treated samples to that of untreated samples in each assay. Double asterisks (**) indicate a significant difference (*P* < 0.01) compared with the stress-treated samples (Student’s *t* test)

To test whether the expression of Lip40 protein in the outer-membrane fraction is stimulated by stress conditions, *A. pleuropneumoniae* cells cultured under anaerobic condition or high and low temperatures were collected, and their outer-membrane fractions were extracted and quantified. Equal amounts of samples (66 μg) were separated by SDS-PAGE, and the expression levels were determined with western blotting (Fig. 5). In general, the expression of Lip40 protein was enhanced under stress conditions, especially at 42 °C, and the Lip40 signal increased more than three-fold under heat shock. These results indicate that both the transcription of the *lip40* gene and the translation of the Lip40 protein increased in response to stress. Therefore, Lip40 may be involved in the adaptation of *A. pleuropneumoniae* to environmental changes.

### Lip40 is protective in mice

**Fig. 6:**
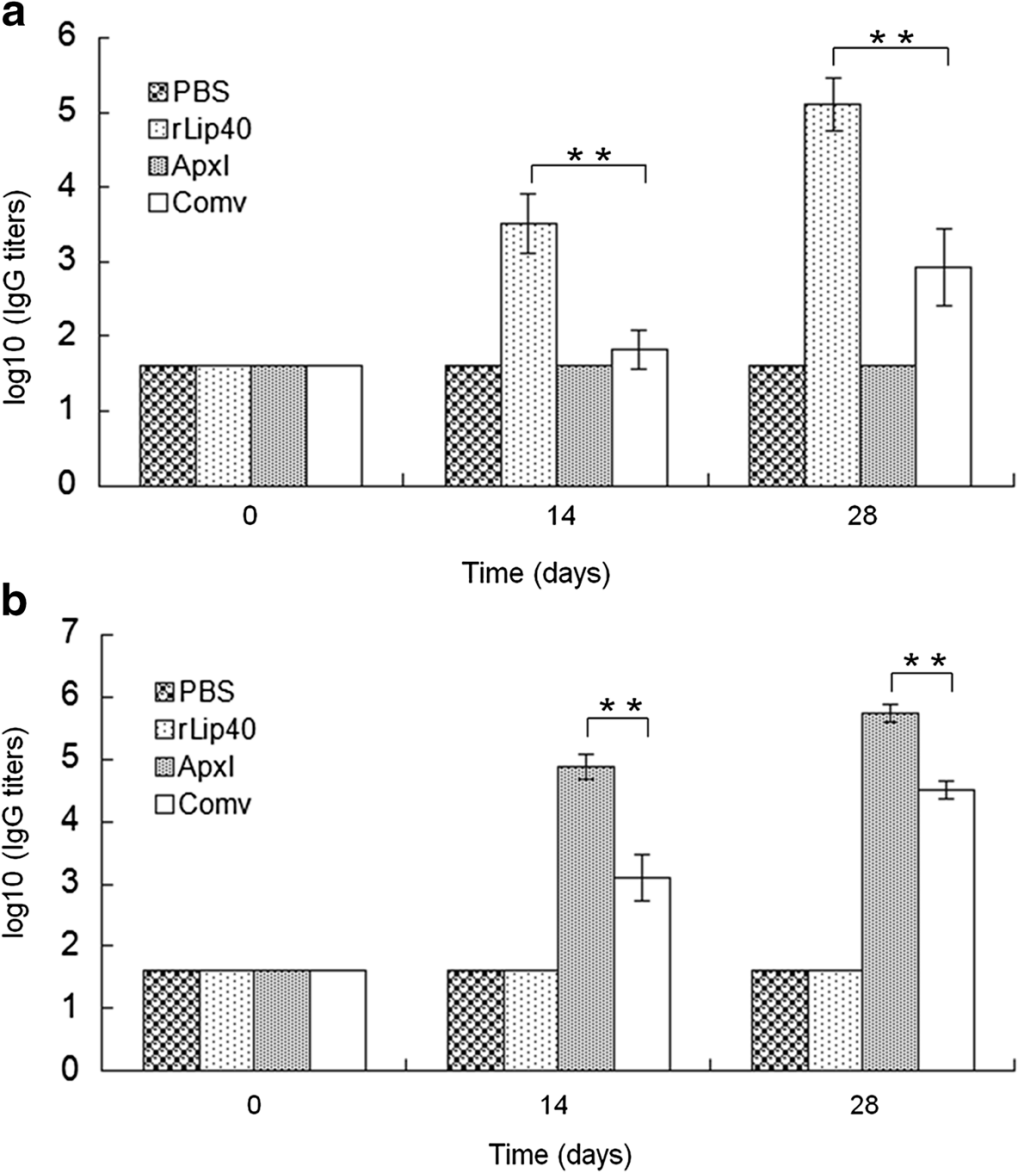
Humoral responses in mice. Antibodies against rLip40 (**a**) and ApxI (**b**) were evaluated at different time points with ELISAs. ELISA titers are expressed as the logarithm (log10) of the reciprocal of the highest dilution of serum with an OD_630_ value above that of the cutoff value for each mouse. Double asterisks (**) indicate *P* < 0.01, calculated with Student’s *t* test

**Fig. 7:**
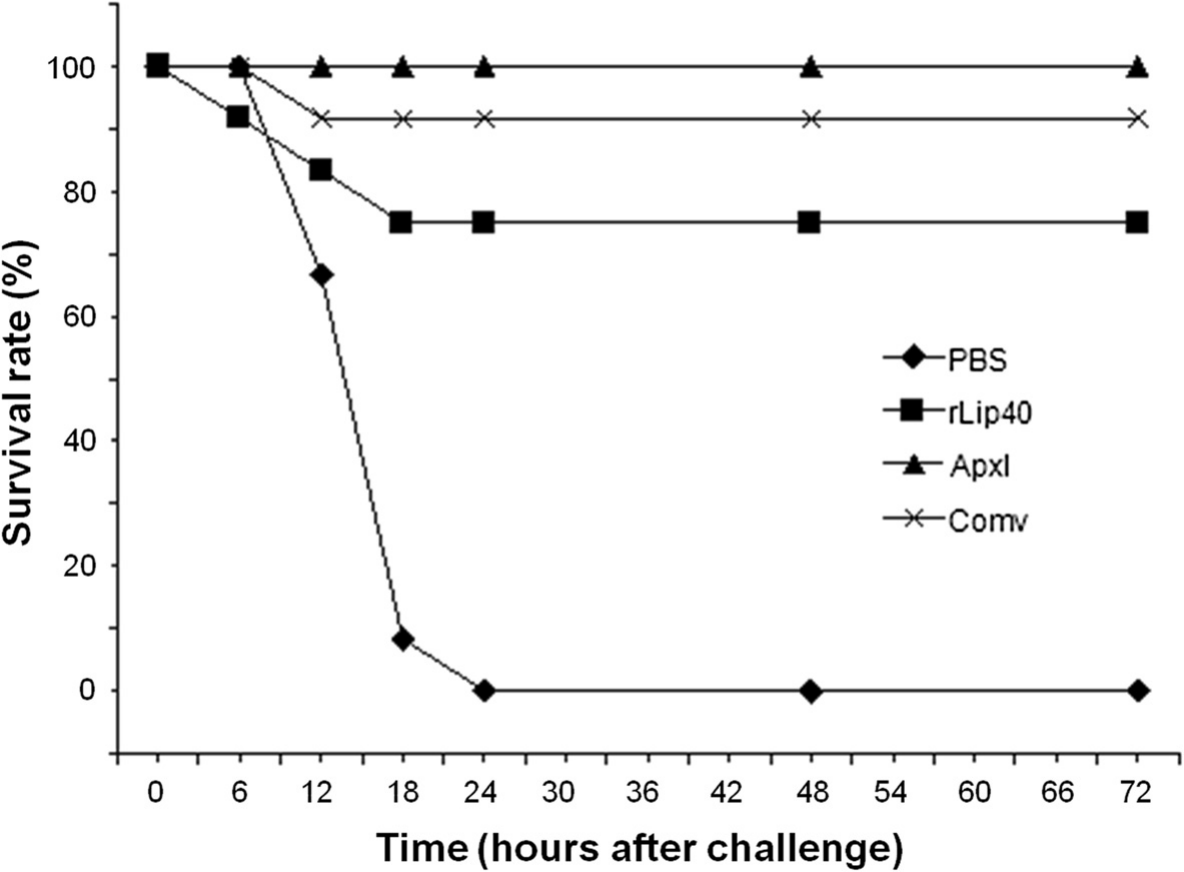
Protection conferred by different antigens against *A. pleuropneumoniae* challenge in mice (survival rates). Numbers of surviving mice in each group was monitored for 5 days after challenge. Numbers of surviving mice did not change 72 h after infection

Serum samples from mice immunized with different antigens were analyzed with ELISAs (Fig. 6). All the mice were negative for rLip40 and ApxI before immunization. Serum samples from the mice inoculated with PBS (group 1) or ApxI (group 3) showed no detectable antibody against Lip40 during this study (Fig. 6a), whereas the sera from group 2, vaccinated with rLip40, were positive for Lip40 antibodies 14 days after the first vaccination, and the titer had increased by the second vaccination. The Lip40 antibody titers in group 2 were significantly higher than those in the other groups (*P* <0.01, Student’s *t* test; Fig. 6a). The mice in groups 1 and 2 were also free of ApxI antibodies, but the mice in group 3 showed a significantly stronger ApxI antibody response than the other groups (*P* < 0.01). As shown in Fig. 6b, group 4, vaccinated with commercial vaccine, also showed a strong ApxI antibody response after vaccination. The mice were challenged with virulent *A. pleuropneumoniae* SLW01 14 days after the second immunization, and the survival rates were recorded for 5 days after challenge (Fig. 7). No mice inoculated with PBS survived the challenge, and the protective efficacies of rLip40, ApxI, and commercial vaccine were 75 % (9/12), 100 % (12/12), and 91.7 % (11/12), respectively. These results show that rLip40 protected more than half the animals (75 %) from the challenge. Thus, the protective efficacy of rLip40 was significantly higher than that of the negative control (0 %), indicating that rLip40 is immunoprotective and provides effective protection against *A. pleuropneumoniae* infection in mice.

## Discussion

Lipoproteins are required for the virulence of some pathogenic bacteria. They play a variety of roles in host–pathogen interactions, from surface adhesion and the initiation of inflammatory processes to the translocation of virulence factors into the host cytoplasm [8]. However, the pathogenesis of *A. pleuropneumoniae* lipoproteins has rarely been examined. Most such studies have focused on their immunogenicity and vaccine potential, including studies of TbpB [26–28] and OmlA [29, 30]. These proteins were shown to be immunoprotective and important vaccine components (PleuroStar APP, Novartis Animal Health Inc., Switzerland). However, PalA was reported to inhibit the protective efficacy of ApxI and ApxII [24].

In the present study, a putative lipoprotein, Lip40, which contains an interesting tandemly repeated sequence, Q(E/D/P)QPK, was characterized. Previous studies have demonstrated the relationship between coding tandem repeat (CTR) sequences and virulence in *Chlamydia pecorum* [31, 32]. CTRs at the *incA* locus encode a variable number of repeated motifs, and the number of repetitions in *incA* is related to the virulence of *C. pecorum* strains [32]. Similarly, the number of tandem repeats in Lip40 varies within its homologues in different serovars of *A. pleuropneumoniae* strains. Therefore, the possible role of Lip40 in bacterial pathogenesis requires further characterization.

Oxygen deprivation and high temperature are two common stresses that *A. pleuropneumoniae* encounters during infection [5, 33]. Transcriptional profiles under anaerobic conditions [33] and acute disease [34] have been analyzed with microarray chips to discover potential virulence factors, which are usually considered to be upregulated under *in vivo* conditions or in a mimicked infection. This genome-wide transcriptional screening of the differentially expressed genes of *A. pleuropneumoniae* revealed no Lip40 homologue. In this study, we confirmed that Lip40 is upregulated at both the transcriptional and translational levels by stress. The mechanisms involved in the adaptive responses of *A. pleuropneumoniae* remain to be investigated. The effects of cold shock were tested as a typical environmental stress. Reports have suggested that the autumn–winter transition is one of the outbreak peaks of porcine pleuropneumonia in China [35], and the environmental temperature is always far lower than the normal growth temperature for mesophilic bacteria during this period. It is possible that the adaptation of *A. pleuropneumoniae* to cold stress is responsible for the infections observed at this time of year. Our results indicate that both the transcriptional and translational levels of Lip40 were elevated at 16 °C compared with those at 37 °C, suggesting that Lip40 is a factor in the responsiveness of *A. pleuropneumoniae* to low temperature. Therefore, we hypothesize that Lip40 is a potential virulence factor and involved in pathogen–environment and/or pathogen–host interactions.

Commercial vaccines, including whole-cell bacterins and second-generation subunit vaccines, only confer partial protection against *A. pleuropneumoniae* infection [29]. Therefore, it is essential that more effective vaccines be developed. The identification of conserved immunogenic antigens, especially outer-membrane-located proteins, will advance this purpose. Several outer-membrane-associated proteins have been verified separately as vaccine components or candidate vaccines with various investigation methods, including TbpB, OmlA, OmpP2, OmpA, and OmpW [25, 36, 37]. In addition to their location on the outer bacterial membrane, many lipoproteins of Gram-negative bacteria are considered to be efficient candidate vaccines [38]. The putative outer-membrane proteins and lipoproteins have been identified in the genome of *Pasteurella multocida*, a great step forward in the development of a protective vaccine against fowl cholera [39, 40]. Similar research was undertaken in *A. pleuropneumoniae*, and several lipoproteins were cloned and their protective efficacies were investigated [25]. Here, we report a putative lipoprotein, Lip40, confirmed to be an outer-membrane-localized stress-responsive factor. Purified rLip40 induced strong antibody responses when it was administered to rabbits. Mice vaccinated with rLip40 displayed a survival rate of 75 %, which was significantly higher than that of the negative control group (0 %), indicating that rLip40 can be used as a component of vaccine against *A. pleuropneumoniae* infection. Our results provide additional confirmation of the importance of bacterial outer-membrane lipoproteins in vaccine development.

## Conclusions

A putative lipoprotein, Lip40, from *A. pleuropneumoniae* was characterized and confirmed to be both immunogenic and localized to the outer membrane. Its expression is responsive to temperature and anaerobic stimuli. Lip40 was also shown to be a potential protective antigen for vaccine development. Further investigation of the roles of Lip40 in *A. pleuropneumoniae* infection should provide insight into the pathogenesis of this bacterium.

## Additional files

Additional file 1: Table S1. Prediction of the potential lipoproteins (LPPs) of *A. pleuropneumoniae (DOC 179 kb)*
Additional file 2: Figure S1. Prevalence of *A. pleuropneumoniae* Lip40 homologues in other Gram-negative bacteria. Multiple sequence alignment was constructed with BioEdit (version 7.0). The conserved amino acid residues are shaded in red. Lipoproteins (LPPs) in *Aggregatibacter actinomycetemcomitans* (YP_004948053.1) and *Cardiobacterium hominis* (ZP_05705901.1) were used in the alignment analysis. (TIFF 2102 kb) Additional file 3: Figure S2. Multiple sequence alignment. Homology between *A. pleuropneumoniae* Lip40 and Tbp proteins in *Haemophilus sputorum* (WP_007525245.1), *Haemophilus pittmaniae* (WP_007242241.1), and *Neisseria sicca* (WP_003766480.1) was analyzed with BioEdit (version 7.0). The conserved amino acid residues are shaded in red. (TIFF 5206 kb) Additional file 4: Figure S3. Evaluation of polyclonal anti-Lip40 antibody. (A) Dynamics of the antibody responses elicited by rLip40. Serum samples were taken from rabbits before each immunization and 10 days after the last immunization. Serum samples were diluted by PBST (1:2000, v/v) and evaluated by Lip40-ELISA. Results indicate the average OD_630_ values. (B) ELISA reactivity of anti-sera against rLip40 and GST. Anti-sera were collected 10 days after the last immunization, serially diluted and evaluated by Lip40-ELISA and GST-ELISA, separately. Results indicate the average OD_630_ values. (TIFF 2703 kb)
