# Supplementary material for: Identification and characterization of a novel stress-responsive outer membrane protein Lip40 from Actinobacillus pleuropneumoniae
Source: BMC Biotechnol. 2015 Nov 25;15:106. doi: 10.1186/s12896-015-0199-8 (PMC4660844; doi:10.1186/s12896-015-0199-8)
Supplement: Additional file 1: Table S1. — Prediction of the potential lipoproteins (LPPs) of A. pleuropneumoniae (DOC 179 kb) [file 12896_2015_199_MOESM1_ESM.doc]

***Table S1*** *Prediction of the potential lipoproteins (LPPs) of* A. pleuropneumoniae

| Locus | Name | PrositeScan | | DOLOP | SignalP analysis | | PrediSi | | Phobius | | LipoP analysis | | | | Verdict |
| --- | --- | --- | --- | --- | --- | --- | --- | --- | --- | --- | --- | --- | --- | --- | --- |
|  |  | Y/N | Cys  position | Y/N | D value | Signal peptide (Y/N) | Score | Signal peptide (Y/N) | End of H-region | End of C-region | Best prediction | Score | Margin | 2nd choice |  |
| APJL_0037 | - | Y | 17 | Y | 0.601 | Y | 0.6982 | Y | 14 | 18 | SpII | 23.2781 | 13.0101 | SpI | Lpp |
| APJL_0038 | *slyB* | Y | 19 | Y | 0.629 | Y | 0.6680 | Y | 17 | 22 | SpII | 23.0626 | 12.5735 | SpI | Lpp |
| APJL_0100 | *nrfA* | Y | 18 | N | 0.871 | Y | 1.0000 | Y | 19 | 25 | SpI | 23.9312 | 12.6796 | SpII | Lpp |
| APJL_0117 | - | Y | 20 | Y | 0.620 | Y | 0.5394 | Y | 17 | 22 | SpII | 25.8917 | 11.6005 | SpI | Lpp |
| APJL_0126 | - | Y | 20 | Y | 0.618 | Y | 0.6752 | Y | 20 | 24 | SpII | 20.3871 | 4.79537 | SpI | Lpp |
| APJL_0157 | *apbE* | Y | 19 | Y | 0.566 | Y | 0.4383 | Y | 16 | 21 | SpII | 14.7451 | 4.87164 | SpI | Lpp |
| APJL_0221 | - | Y | 19 | Y | 0.607 | Y | 0.5505 | Y | 14 | 19 | SpII | 23.3551 | 14.5504 | SpI | Lpp |
| APJL_0223 | - | Y | 17 | Y | 0.627 | Y | 0.7357 | Y | 14 | 18 | SpII | 22.5587 | 8.45356 | SpI | Lpp |
| APJL_0228 | - | Y | 16 | N | 0.650 | Y | 0.6820 | Y | 12 | 17 | SpII | 22.3809 | 12.5648 | SpI | Lpp |
| APJL_0239 | - | Y | 22 | Y | 0.636 | Y | 0.5721 | Y | 19 | 24 | SpII | 27.8905 | 14.9846 | SpI | Lpp |
| APJL_0250 | *tbpB2* | Y | 20 | Y | 0.520 | Y | 0.4179 | Y | 17 | 22 | SpII | 19.9887 | 2.52507 | SpI | Lpp |
| APJL_0265 | *tolC* | Y | 19 | Y | 0.760 | Y | 0.8193 | Y | 17 | 25 | SpII | 21.436 | 16.0827 | SpI | Lpp |
| APJL_0317 | *palA* | Y | 20 | Y | 0.814 | Y | 0.7777 | Y | 18 | 23 | SpII | 27.8366 | 15.8113 | SpI | Lpp |
| APJL_0347 | - | Y | 20 | Y | 0.626 | Y | 0.6162 | N | 17 | 21 | SpII | 27.1961 | 10.5153 | SpI | Lpp |
| APJL_0348 | *hlpB* | Y | 20 | Y | 0.789 | Y | 0.9368 | Y | 15 | 19 | SpII | 18.3233 | 15.1906 | SpI | Lpp |
| APJL_0373 | - | Y | 19 | Y | 0.578 | Y | 0.6457 | Y | 16 | 20 | SpII | 14.3932 | 8.99979 | SpI | Lpp |
| APJL_0386 | *potD* | Y | 20 | Y | 0.631 | Y | 0.7964 | Y | 15 | 20 | SpII | 16.7855 | 9.29752 | SpI | Lpp |
| APJL_0410 | *ompP4* | Y | 20 | Y | 0.733 | Y | 0.5716 | Y | 17 | 21 | SpII | 19.2404 | 11.1444 | SpI | Lpp |
| APJL_0453 | *smpA* | Y | 19 | Y | 0.578 | Y | 0.5811 | Y | 14 | 19 | SpII | 16.3647 | 10.2409 | SpI | Lpp |
| APJL_0487 | *plpD* | Y | 22 | Y | 0.797 | Y | 0.5926 | Y | 12 | 18 | SpII | 14.6432 | 8.6443 | SpI | Lpp |
| APJL_0542 | *tadD* | Y | 21 | Y | 0.703 | Y | 0.7478 | Y | 16 | 21 | SpII | 18.2711 | 7.80173 | SpI | Lpp |
| APJL_0586 | - | Y | 15 | Y | 0.637 | Y | 0.6215 | Y | 18 | 23 | SpI | 9.45955 | -0.0202 | SpII | Lpp |
| APJL_0605 | - | Y | 24 | Y | 0.636 | Y | 0.5431 | Y | 21 | 25 | SpII | 16.3401 | 2.99238 | SpI | Lpp |

| Locus | Name | PrositeScan | | DOLOP | SignalP analysis | | PrediSi | | Phobius | | LipoP analysis | | | | Verdict |
| --- | --- | --- | --- | --- | --- | --- | --- | --- | --- | --- | --- | --- | --- | --- | --- |
|  |  | Y/N | Cys  position | Y/N | D value | Signal peptide (Y/N) | Score | Signal peptide (Y/N) | End of H-region | End of C-region | Best prediction | Score | Margin | 2nd choice |  |
| APJL_0633 | - | Y | 19 | Y | 0.569 | Y | 0.3382 | N | 16 | 21 | SpII | 21.9021 | 4.29373 | SpI | Lpp |
| APJL_0780 | *lolB* | Y | 19 | Y | 0.623 | Y | 0.6341 | Y | 19 | 24 | SpII | 19.8965 | 12.5512 | SpI | Lpp |
| APJL_0822 | *mltA* | Y | 22 | Y | 0.521 | Y | 0.3188 | Y | 19 | 24 | SpII | 14.3893 | 2.11782 | SpI | Lpp |
| APJL_0885 | *lptE* | Y | 16 | Y | 0.527 | Y | 0.1296 | Y | 13 | 17 | SpII | 14.6801 | -0.2009 | CYT | Lpp |
| APJL_0921 | *plpB* | Y | 20 | Y | 0.424 | Y | 0.2061 | Y | 17 | 25 | SpII | 20.2166 | 2.80067 | SpI | Lpp |
| APJL_0922 | *hlpA* | Y | 20 | Y | 0.397 | Y | 0.3119 | Y | 17 | 22 | SpII | 22.6735 | 4.28955 | SpI | Lpp |
| APJL_0931 | - | Y | 21 | Y | 0.517 | Y | 0.3035 | Y | 18 | 23 | SpII | 22.9782 | 5.52076 | SpI | Lpp |
| APJL_0973 | - | Y | 17 | Y | 0.656 | Y | 0.5389 | Y | 14 | 20 | SpII | 11.5529 | 4.49911 | SpI | Lpp |
| APJL_1078 | - | Y | 22 | Y | 0.718 | Y | 0.6801 | Y | 15 | 20 | SpII | 22.4242 | 9.34104 | SpI | Lpp |
| APJL_1140 | - | Y | 20 | Y | 0.736 | Y | 0.6931 | Y | 18 | 23 | SpII | 22.0917 | 14.9426 | SpI | Lpp |
| APJL_1172 | - | Y | 19 | Y | 0.706 | Y | 0.8805 | Y | 17 | 22 | SpII | 28.6 | 19.1669 | SpI | Lpp |
| APJL_1178 | - | Y | 20 | Y | 0.497 | Y | 0.3839 | Y | 17 | 21 | SpII | 27.4499 | 9.45505 | SpI | Lpp |
| APJL_1284 | *pilF* | Y | 22 | N | 0.611 | Y | 0.5444 | Y | 20 | 24 | SpII | 19.9876 | 4.08837 | SpI | Lpp |
| APJL_1310 | - | Y | 19 | Y | 0.558 | Y | 0.7145 | Y | 16 | 21 | SpII | 23.8663 | 12.4153 | SpI | Lpp |
| APJL_1318 | - | Y | 17 | Y | 0.629 | Y | 0.6965 | Y | 15 | 20 | SpII | 24.0194 | 9.3078 | SpI | Lpp |
| APJL_1380 | - | Y | 25 | Y | 0.609 | N | 0.5356 | Y | 22 | 27 | SpII | 12.1035 | 4.75414 | SpI | Lpp |
| APJL_1383 | - | Y | 23 | N | 0.718 | Y | 0.5515 | Y | 20 | 32 | SpII | 15.7304 | 7.86534 | SpI | Lpp |
| APJL_1429 | - | Y | 23 | Y | 0.528 | Y | 0.2223 | N | 19 | 23 | SpII | 12.5833 | 1.64909 | SpI | Lpp |
| APJL_1467 | - | Y | 19 | Y | 0.683 | Y | 0.8548 | Y | 14 | 19 | SpII | 27.3061 | 14.1635 | SpI | Lpp |
| APJL_1469 | - | Y | 19 | Y | 0.525 | Y | 0.7149 | Y | 15 | 19 | SpII | 19.4116 | 4.98437 | SpI | Lpp |
| APJL_1502 | - | Y | 19 | N | 0.599 | Y | 0.4701 | Y | 17 | 22 | SpII | 16.264 | 6.94836 | SpI | Lpp |
| APJL_1607 | - | Y | 17 | Y | 0.670 | Y | 0.6058 | Y | 15 | 19 | SpII | 18.8835 | 10.069 | SpI | Lpp |
| APJL_1615 | *cpxD* | Y | 22 | Y | 0.593 | Y | 0.8029 | Y | 21 | 33 | SpII | 21.2449 | 13.0256 | SpI | Lpp |
| APJL_1666 | - | Y | 20 | Y | 0.705 | Y | 0.7214 | Y | 17 | 22 | SpII | 21.3007 | 9.31711 | SpI | Lpp |
| APJL_1726 | - | Y | 23 | Y | 0.706 | Y | 0.7056 | Y | 20 | 25 | SpII | 20.0759 | 12.1016 | SpI | Lpp |

| Locus | Name | PrositeScan | | DOLOP | SignalP analysis | | PrediSi | | Phobius | | LipoP analysis | | | | Verdict |
| --- | --- | --- | --- | --- | --- | --- | --- | --- | --- | --- | --- | --- | --- | --- | --- |
|  |  | Y/N | Cys  position | Y/N | D value | Signal peptide (Y/N) | Score | Signal peptide (Y/N) | End of H-region | End of C-region | Best prediction | Score | Margin | 2nd choice |  |
| APJL_1740 | *tolA2* | Y | 19 | Y | 0.688 | Y | 0.7076 | Y | 16 | 20 | SpII | 29.7254 | 12.2591 | SpI | Lpp |
| APJL_1777 | *mltC* | Y | 20 | Y | 0.658 | Y | 0.4624 | Y | 18 | 23 | SpII | 18.4597 | 4.31175 | SpI | Lpp |
| APJL_1783 | *omlA* | Y | 20 | Y | 0.612 | Y | 0.7144 | Y | 18 | 30 | SpII | 29.8829 | 11.6926 | SpI | Lpp |
| APJL_1919 | - | Y | 20 | Y | 0.650 | Y | 0.6948 | Y | 20 | 24 | SpII | 22.7824 | 10.3697 | SpI | Lpp |
| APJL_1942 | - | Y | 20 | Y | 0.654 | Y | 0.5802 | Y | 18 | 23 | SpII | 22.8934 | 9.8269 | SpI | Lpp |
| APJL_1960 | *pepO* | Y | 19 | Y | 0.594 | Y | 0.5946 | Y | 16 | 21 | SpII | 23.2649 | 12.2627 | SpI | Lpp |
| APJL_1965 | *vacJ* | Y | 18 | Y | 0.580 | Y | 0.5640 | Y | 16 | 21 | SpII | 21.484 | 9.10596 | SpI | Lpp |
| APJL_1976 | - | Y | 21 | Y | 0.661 | Y | 0.8974 | Y | 19 | 31 | SpII | 32.3619 | 20.7824 | SpI | Lpp |
| APJL_1977 | - | Y | 17 | Y | 0.753 | Y | 0.7756 | Y | 14 | 19 | SpII | 23.4243 | 13.5267 | SpI | Lpp |
| APJL_2004 | - | Y | 21 | Y | 0.587 | N | 0.5666 | N | 18 | 23 | SpII | 23.5947 | 5.20767 | SpI | Lpp |
| APJL_2060 | *hbpA2* | Y | 22 | Y | 0.670 | Y | 0.5974 | Y | 17 | 22 | SpII | 24.5797 | 6.52646 | SpI | Lpp |
| APJL_2094 | - | Y | 16 | Y | 0.619 | Y | 0.6451 | Y | 14 | 19 | SpII | 17.2059 | 10.8079 | SpI | Lpp |
