# Supplementary figures and images for: Identification and characterization of a novel stress-responsive outer membrane protein Lip40 from Actinobacillus pleuropneumoniae
Source: BMC Biotechnol. 2015 Nov 25;15:106. doi: 10.1186/s12896-015-0199-8 (PMC4660844; doi:10.1186/s12896-015-0199-8)

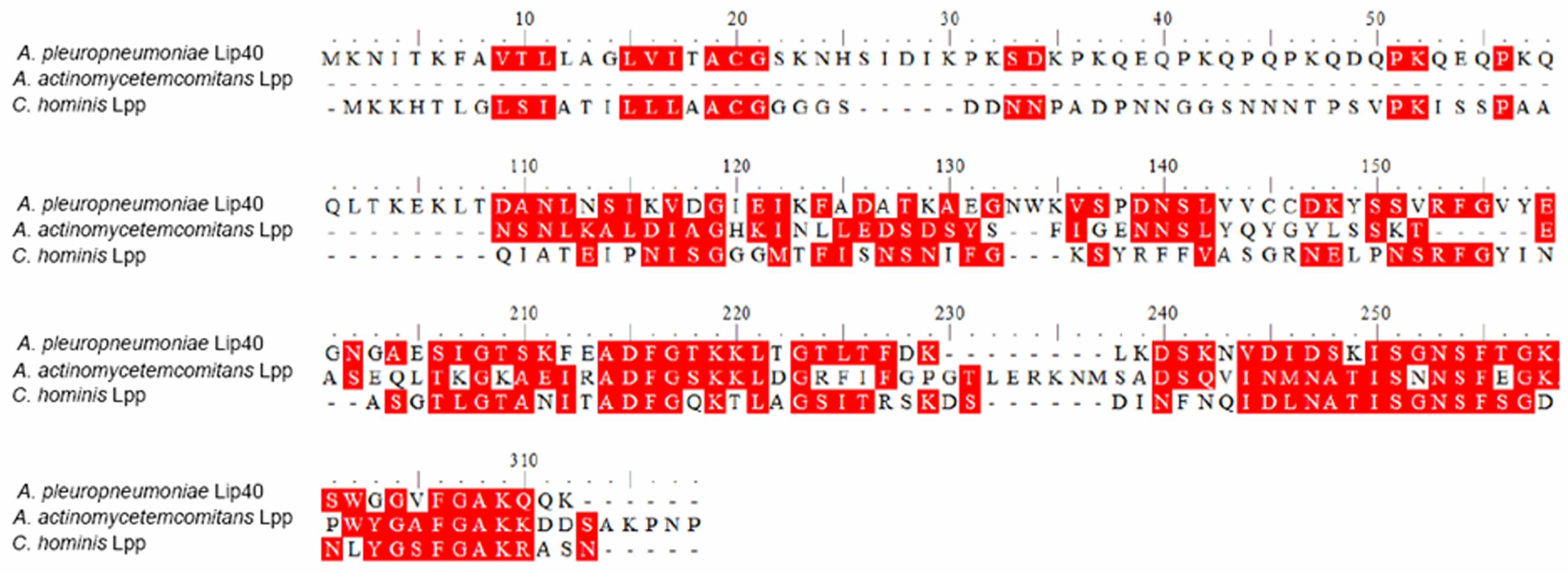

Supplement: Additional file 2: Figure S1. — Prevalence of A. pleuropneumoniae Lip40 homologues in other Gram-negative bacteria. Multiple sequence alignment was constructed with BioEdit (version 7.0). The conserved amino acid residues are shaded in red. Lipoproteins (LPPs) in Aggregatibacter actinomycetemcomitans (YP_004948053.1) and Cardiobacterium hominis (ZP_05705901.1) were used in the alignment analysis. (TIFF 2102 kb) [file 12896_2015_199_MOESM2_ESM.tif]

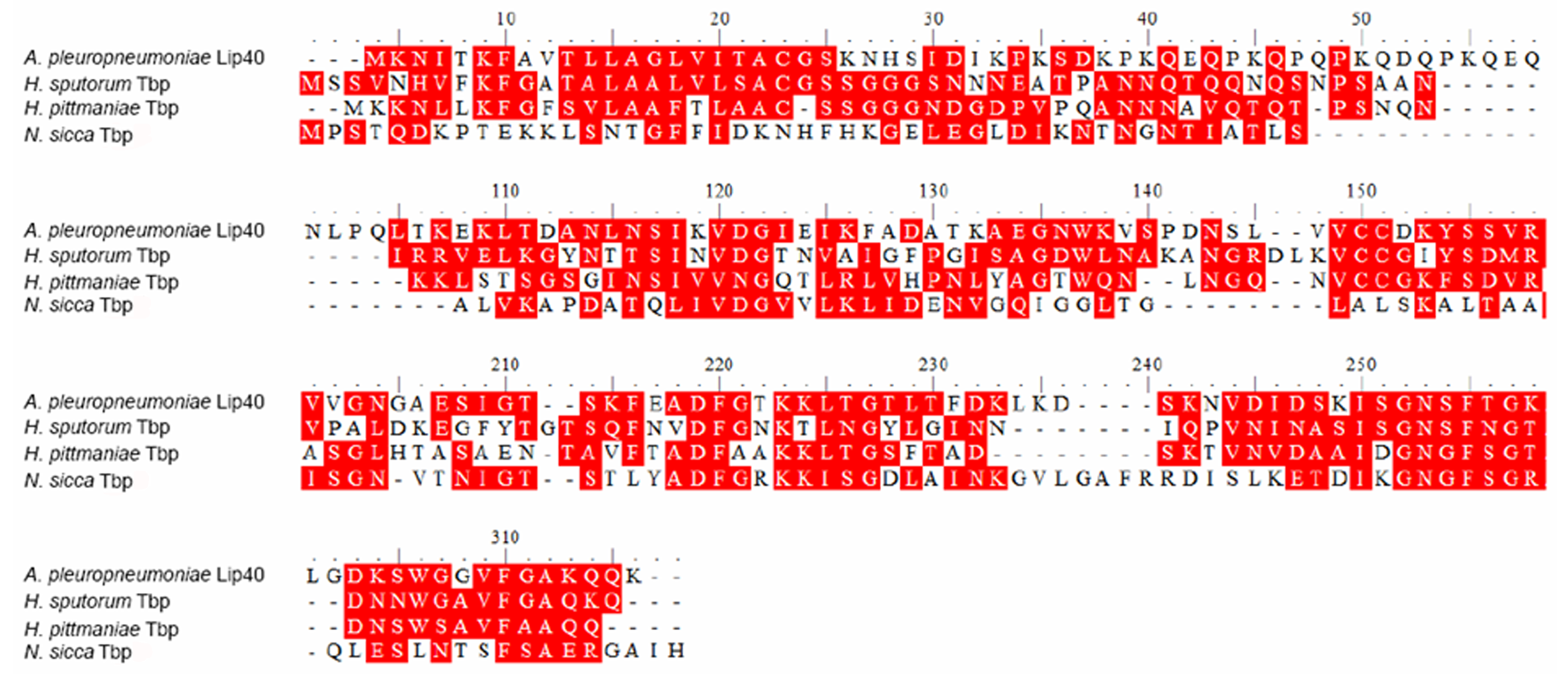

Supplement: Additional file 3: Figure S2. — Multiple sequence alignment. Homology between A. pleuropneumoniae Lip40 and Tbp proteins in Haemophilus sputorum (WP_007525245.1), Haemophilus pittmaniae (WP_007242241.1), and Neisseria sicca (WP_003766480.1) was analyzed with BioEdit (version 7.0). The conserved amino acid residues are shaded in red. (TIFF 5206 kb) [file 12896_2015_199_MOESM3_ESM.tif]

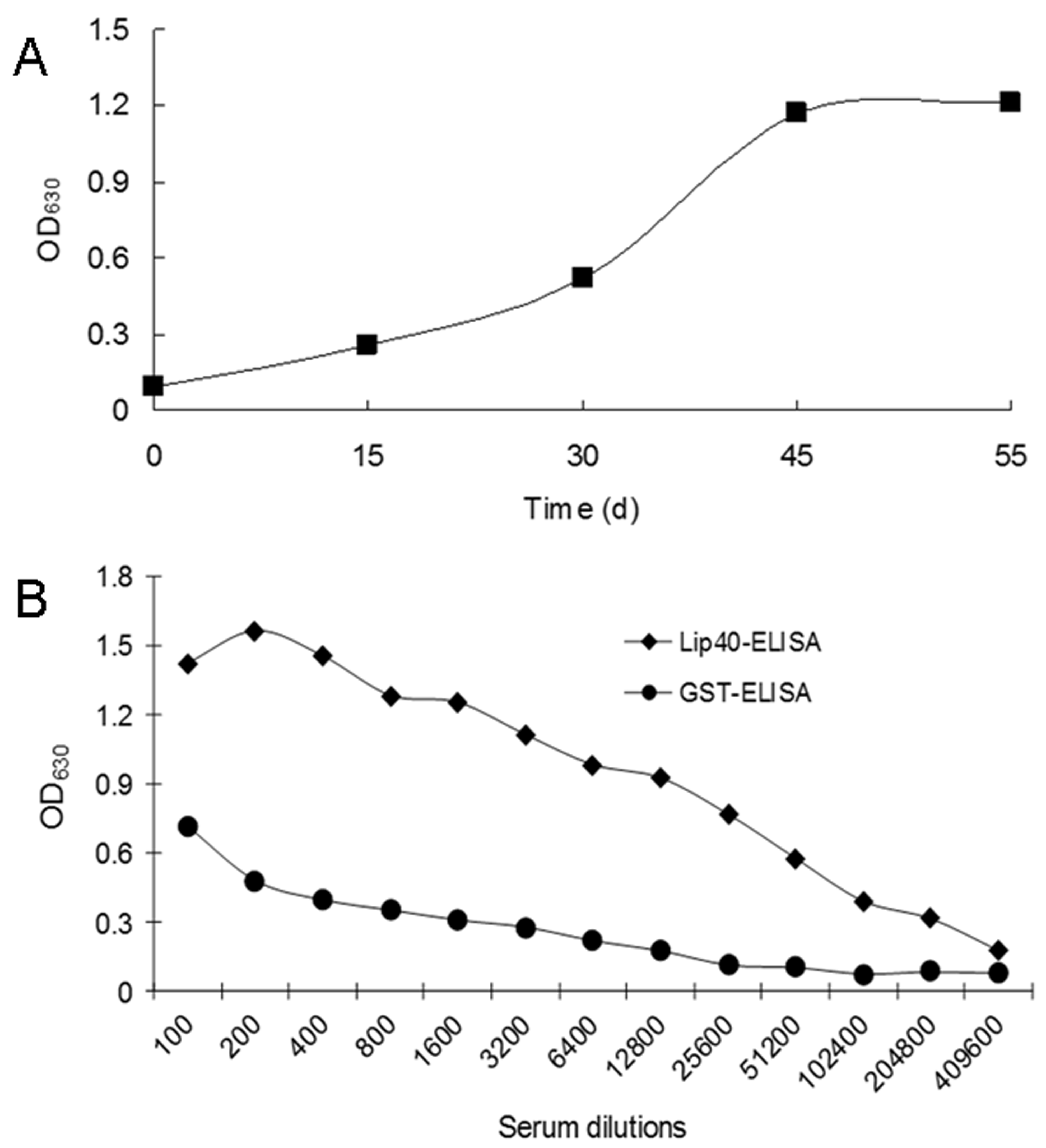

Supplement: Additional file 4: Figure S3. — Evaluation of polyclonal anti-Lip40 antibody. (A) Dynamics of the antibody responses elicited by rLip40. Serum samples were taken from rabbits before each immunization and 10 days after the last immunization. Serum samples were diluted by PBST (1:2000, v/v) and evaluated by Lip40-ELISA. Results indicate the average OD630 values. (B) ELISA reactivity of anti-sera against rLip40 and GST. Anti-sera were collected 10 days after the last immunization, serially diluted and evaluated by Lip40-ELISA and GST-ELISA, separately. Results indicate the average OD630 values. (TIFF 2703 kb) [file 12896_2015_199_MOESM4_ESM.tif]
